# Pacing and energy management with digital tools for conditions with chronic fatigue: What we know so far in ME/CFS and Long COVID

**DOI:** 10.1371/journal.pdig.0001586

**Published:** 2026-07-27

**Authors:** Nilihan E. M. Sanal-Hayes, Lawrence D. Hayes, Marie Mclaughlin, Nicholas F. Sculthorpe

**Affiliations:** 1 Directoriate of Psychology and Sport, School of Health and Society, University of Salford, Salford, United Kingdom; 2 Lancaster Medical School, Faculty of Health and Medicine, Lancaster University, Lancaster, United Kingdom; 3 School of Education and Sport, University of Edinburgh, Edinburgh, United Kingdom; 4 Sport and Physical Activity Research Institute, School of Health and Life Sciences, University of the West of Scotland, Glasgow, United Kingdom; University of Vic - Central University of Catalonia, SPAIN

## Why pacing matters in conditions with post‑exertional symptom exacerbation

‘Pacing’, or ‘energy management’, is a structured approach to regulating one’s physical, cognitive, and emotional activities to remain within limits imposed by chronic illnesses such as fibromyalgia, cancer, ME/CFS, and Long COVID [1,2]. Pacing ostensibly prevents post‑exertional symptom exacerbation, notably post‑exertional malaise (PEM), the worsening of fatigue, pain, cognitive dysfunction, and other symptoms following even modest activity. Across the literature, pacing is heterogeneously described as strategies that include activity planning, routine‑setting, pre‑emptive rest, breaking tasks into smaller components, monitoring energy fluctuations, and intentionally avoiding activity “push‑crash” cycles that commonly drive clinical deterioration [3]. In addition to heterogenous implementation, evidence of efficacy is also varied [3–9]. In this article, we will focus on ME/CFS and Long COVID as use case examples.

Historically, pacing emerged as an energy management method encouraging individuals to do as much as they can within their limits, rather than pushing through symptoms or following externally prescribed exercise. The central principle is that of the “energy envelope” [1], which asks individuals to identify the threshold at which activity begins to provoke symptoms and then remain inside that envelope as consistently as possible.

While definitions vary across clinicians, researchers, and patient groups, a consistent theme in both our systematic review and the scoping review is that pacing is individualised and requires ongoing adjustment as symptoms fluctuate [3,7]. Importantly, pacing is now the only management strategy recommended by the 2021 NICE guidelines for ME/CFS [10], underscoring its centrality in clinical care despite the concerning lack of evidence for efficacy. As Long COVID research evolved, pacing increasingly became recognised as a pragmatic, do no harm approach for a condition characterised by varied symptomology, unpredictable recovery patterns, and vulnerability to overexertion [8,11].

## What digital tools offer

Modern wearables such as smart watches, rings, and phones can record pulse rate, step count, and pulse rate variability [12,13]. When data streams are coupled with a smartphone app, they can provide in the moment prompts that nudge users to slow down before they cross a threshold associated to symptom exacerbation. This is the core concept behind just‑in‑time adaptive interventions for pacing [6,14]. Real time symptom tracking, for example, through short in‑app check‑ins or ecological momentary assessments, captures fluctuations in symptoms that traditional pre- and post-study measures miss [15]. Higher frequency sampling helps individuals and clinicians identify idiosyncratic triggers such as cognitive loads, temperature, or specific activity intensities, and adjust plans accordingly. People with severe ME/CFS or those housebound with Long COVID often cannot attend in‑person appointments or laboratory visits. Smartphones act as a lab in the pocket by combining sensors and user input, enabling scalable support for populations that are commonly underserved, such as people in remote locations [16]. However, the digital divide may exacerbate these issues and older patients, those with low digital literacy, or those in lower-income settings may face substantial barriers to engaging with wearable-based pacing tools [17].

## The PaceMe app: What we learned from a randomised controlled trial in Long COVID

In our recent 6-month randomised controlled trial, we examined a pacing platform, PaceMe, that combined a wearable activity tracker with just‑in‑time messages based on a personalised activity allowance [6]. Participants received notifications at 50%, 75%, and 100% of their daily allowance, initially defined using pulse rate zones and adjusted over time based on PEM reports. The primary outcome was PEM using the DePaul Symptom Questionnaire-PEM (DSQ-PEM). The intervention was not superior to usual care.

While it may be tempting to conclude that digital pacing is ineffective. We propose that, for certain individuals, particularly given our inclusion criteria of symptoms lasting longer than 4 weeks, Long COVID may exhibit gradual improvement over several months (which we have previously demonstrated in other digital symptom tracking work [15]). This phenomenon is distinct from ME/CFS, as we have previously demonstrated in other studies [15]. Such improvement could obscure differences between groups in pacing trials that focus on PEM. Conversely, it is equally plausible that there is a null effect of digital energy management on PEM frequency and severity. However, our intervention was safe and feasible, with no adverse events, and qualitative feedback indicated users felt greater insight and control [18]. For future research, we recommend testing the same framework in conditions without expected recovery, such as ME/CFS [19]. For clinicians working with people with chronic fatigue, we have shown wearable-based pacing appears safe and feasible, that users report perceived benefit, and it warrants further investigation in conditions without expected natural recovery.

## Challenges in operationalising digital pacing

For many, biometrics such as pulse rate provide an early warning that effort is transitioning into a zone associated with adverse symptoms. Setting conservative thresholds, for example, limiting time above a personalised heart rate, can help maintain activity within one’s envelope. Currently, this requires manual input from the patient to programme their device to give alerts at this heart rate, as most smart devices exist to encourage a gradual increase in activity.

Despite the benefits of passive data collection and digitisation, the ability to capture a holistic energy expenditure remains elusive and still requires user input. A potential area for harm is data overload, where many prompts or alerts can worsen symptoms through overstimulation or fixation. Limiting notification frequency and ensuring quiet hours (“do not disturb”) reduces burden and may increase adherence.

## Bottom line

Pacing works best when it is personalised, conservative, and supported by timely feedback. Digital tools can measure exertion in the background and nudge people before they cross their own thresholds. We reported safety and feasibility in people with Long COVID, user perceived value, but no added benefit over usual care for PEM over 6 months. Rather than a dead end, this is a map for refinement and for targeted testing in ME/CFS, where limited natural recovery creates a clearer signal to detect.
